# Multiple Poliovirus Proteins Repress Cytoplasmic RNA Granules

**DOI:** 10.3390/v7122922

**Published:** 2015-12-25

**Authors:** Jonathan D. Dougherty, Wei-Chih Tsai, Richard E. Lloyd

**Affiliations:** Department of Molecular Virology and Microbiology, Baylor College of Medicine, Houston, TX 77030, USA; Wei-Chih.Tsai@bcm.edu

**Keywords:** stress granule, processing body, P-body, G3BP1, Tia1, poliovirus, Rck/p54, J0101

## Abstract

We have previously shown that poliovirus (PV) infection induces stress granule (SG) formation early in infection and then inhibits the formation of SG and disperses processing bodies (PBs) by the mid-phase of infection. Loss of SG was linked to cleavage of G3BP1 by viral 3C proteinase (3C^pro^), however dispersal of PBs was not strongly linked to cleavage of specific factors by viral proteinases, suggesting other viral proteins may play roles in inhibition of SG or PB formation. Here we have screened all viral proteins for roles in inducing or inhibiting the formation of RNA granules by creating fusions with mCherry and expressing them individually in cells. Expression of viral proteins separately revealed that the capsid region P1, 2A^pro^, 3A, 3C^pro^, the protease precursor 3CD and 3D polymerase all affect RNA granules to varying extents, whereas 2BC does not. 2A^pro^, which cleaves eIF4GI, induced SGs as expected, and entered novel foci containing the SG nucleating protein G3BP1. Of the two forms of G3BP, only G3BP1 is cleaved by a virus proteinase, 3C^pro^, whereas G3BP2 is not cleaved by 3C^pro^ or 2A^pro^. Surprisingly, 3CD, which contains proteinase activity, differentially repressed PBs but not SGs. Further, both 2A^pro^ and 3C^pro^ expression dispersed PBs, however molecular targets were different since PB dispersal due to 2A^pro^ and heat shock protein (Hsp)90 inhibition but not 3C^pro^, could be rescued by application of oxidative stress to cells. The data indicate that PV repression of SGs and PBs is multifactorial, though protease function is dominant.

## 1. Introduction

Somatic cells form two major classes of cytoplasmic RNA granules known as stress granules (SGs) and processing bodies (PBs) that transiently store silenced messenger ribonucleoproteins (mRNPs). Stress granules form in response to many types of environmental stress and serve as triage centers for silenced mRNPs. As such, they contain stalled translation initiation complexes consisting of small ribosome subunits, mRNA, translation initiation factors and many RNA binding proteins [1,2,3]. In contrast, PBsare usually constitutively present in cells, however, they also respond to stress with by increasing their size and number. Unlike SGs, PBs contain mRNAs in association with 5′ and 3′-mediated RNA decay factors, mRNA decapping, deadenylation, micro RNA (miRNA)-mediated mRNA silencing, and mRNA storage and are devoid of most translation initiation factors and ribosome subunits [1,4,5]. Both SGs and PBs are very dynamic foci, with protein and mRNA components rapidly exchanging with the surrounding cytosol. Stress granules and PBs are distinguished by their functional protein constituents and key marker proteins. For SGs this includes initiation factors eIF4G, eIF3, eIF4E, small ribosome subunits and SG-nucleating proteins G3BP and Tia-1, among others. PBs are identified by containing RNA helicase Rck/p54 (also known as DDX6), the 5′-3′ exoribonuclease Xrn1, mRNA-decapping enzymes 1a and 2, (Dcp1a and Dcp2), and PABP1-dependent poly(A) nucleases 2 and 3, (Pan2/Pan3) [6].

SGs and PBs function as extensions of translation regulation mechanisms and spatially coordinate networks of mRNPs that are stored or actively translated. This is especially true during non-steady state conditions in the cell. In addition, defects in RNA granule functions have been linked to multiple neurodegenerative, developmental disorders and an increasing role of RNA granules in cancer biology is emerging [7,8,9,10]. Because of the prominence of RNA granules in regulation of gene expression, many viruses are known to antagonize RNA granule function to maintain efficient rates of expression of their proteins and to prevent their genomes and transcripts from being compartmentalized in these silencing compartments [11,12].

We have previously shown that poliovirus (PV) disrupts SG formation by cleavage of the key SG nucleating protein G3BP1 and that PV disperses PBs in conjunction with cleavage and degradation of Dcp1a, Pan3 and Xrn1 [13,14]. Prevention of G3BP cleavage restores SG formation in infected cells suggesting this is a major mechanism of SG disruption, however the mechanism of PB disruption is less clear [14,15]. To further characterize mechanisms of RNA granule disruption by poliovirus we have expressed all the major proteins encoded by the virus individually and determined their effect on modulating both SG and PB assembly. We confirm that PV 2A proteinase (2A^pro)^ expression strongly induces SGs but surprisingly it disperses PBs. 2A^pro^ enters unique cytoplasmic foci containing G3BP1, but does not cleave either G3BP1 or G3BP2 and the 2A-mediated dispersal of PBs could be overcome by exogenous stress in the absence of other viral proteins. In contrast, PB dispersal by 3C proteinase (3C^pro^) could not be countered by exogenous stress and uniquely dispersed PB foci containing ectopic Dcp1a. Other viral polypeptides had little or modest effects on RNA granules, however, the precursor of 3C^pro^, 3CD, surprisingly did not block SG formation but did affect PBs. Overall, the data indicate that 3C^pro^ is the key viral protein that represses both types of cytoplasmic RNA granules. 

## 2. Materials and Methods

### 2.1. Cell Culture, Transfections and Infections

HeLa and A549 cells were used in experiments and grown in Dulbecco’s Modified Eagle’s Media (DMEM) supplemented with 10% fetal bovine serum (FBS) and incubated at 37 °C with 5% CO_2_. For transfections cells were plated at a density of 1.5 × 10^5^ cells/well in 12-well plates and 24 h later cells were transfected with indicated plasmid DNAs using Xtremegene-HP transfection reagent (Roche, Basel, Switzerland) or Lipofectamine 3000 (ThermoFisher, Rockford, IL, USA) according to manufacturer’s protocol. Cells were routinely transfected with 0.5 μg DNA per 1.5 × 10^5^ cells. Poliovirus Type 1 (Mahoney strain) was used to infect cells as indicated at a multiplicity of infection of 10. 

### 2.2. Antibodies

The following antibodies were used in immunoblots and immunofluorescence: rabbit anti-G3BP1 [14], mouse anti-G3BP1 (Bethyl Labs, Montgomery, TX, USA), rabbit anti-G3BP2 (Assay Biotech, Sunnyvale, CA, USA), goat anti-TIA1 (Santa Cruz, Dallas, TX, USA), goat anti-TIAR (Santa Cruz), anti-Rck/p54(DDX6) (gift from CE Cameron), anti-rabbit Alexa Fluor 488 (ThermoFisher, Rockford, IL, USA), anti-mouse Alexa Fluor 647 (Invitrogen), anti-rabbit Texas Red (ThermoFisher), anti-goat Texas Red (ThermoFisher).

### 2.3. Plasmids and Cloning

Plasmids encoding poliovirus protein-mCherry fusions were also created using the In-Fusion system. PCR products were generated from the plasmid pTPOV [16] using the following primers:


P1-Cherry:
 Forward: TGGAATTCTGCAGATATGGGTGCTCAGGTTTCATCACAGAAAGTGGG
 Reverse: GCCACTGTGCTGGATTTATATGTGGTCAGATCCTTGGTGGAGAGGGG
PV 2A-Cherry:
 Forward: TGGAATTCTGCAGAT ATGGGATTCGGACACCAAAACAAAGCGGTG
 Reverse: GCCACTGTGCTGGATTTGCTCCATGGCTTCTTCTTCGTAGGCATACAA
PV 3C-Cherry:
 Forward: TGGAATTCTGCAGATATGGGACCAGGGTTCGATTACGCAGT
 Reverse: GCCACTGTGCTGGATTTTTGACTCTGAGTGAAGTATGATCGC
PV 3CD-Cherry
 Forward: TGGAATTCTGCAGATATGGGACCAGGGTTCGATTACGCAGT
 Reverse: GCCACTGTGCTGGATTTCCGTTGGCTTGACTCATTTTAG
PV 3D-Cherry
 Forward: TGGAATTCTGCAGATATGGGTGAAATCCAGTGGATGAGA
 Reverse: GCCACTGTGCTGGATTTCCGTTGGCTTGACTCATTTTAG
Cherry-PV-Vpg-3C:
 Forward: TGGAATTCTGCAGATACCATGGTGCCCACCATTCGGACAGCAAAGGTACAAGGACCAGGG
 Reverse: GCCACTGTGCTGGATTTATTGACTCTGAGTGAAGTATGATCGCTTCAGGGCCGCTGCAAA
PV 2C-Cherry:
 Forward: TGGAATTCTGCAGATATGGGTGACAGTTGGTTGAAGAAGTTTACTGAAGCATGC
 Reverse: GCCACTGTGCTGGATTTGAAACAAAGCCTCCATACAATTGCCAATGTTGGATCTTCTGTT
PV 2BC-Cherry:
 Forward: TGGAATTCTGCAGATATG GGCATCACCAATTACATAGAGTCACTTGGGGCC
 Reverse: GCCACTGTGCTGGATTTGAAACAAAGCCTCCATACAATTGCCAATGTTGGATCTTCTGTT
PV 3A-Cherry:
 Forward: TGGAATTCTGCAGATATGGGACCACTCCAGTATAAAGACTTGAAAATTGACATCAAGACGAGT
 Reverse: GCCACTGTGCTGGATCTGGTGTCCAGCAAACAGTTTATACATGACATAGAC.


Plasmids PCR amplicons were inserted into vectors pcDNA4/TO-NCherry-mycHisA or pcDNA4/TO-CCherry-mycHisA. These vectors were obtained from T. Albrect and E.J. Wagner (University of Texas Medical Branch, Galveston, TX, USA) and were created from pcDNA4/TO-NCherry-mycHisAby (ThermoFisher) by insertion of mCherry bearing the identical multiple cloning sites in frame upstream or downstream of mCherry.

### 2.4. Immunofluorescence

Cells seeded on glass coverslips and transfected with appropriate plasmids were allowed to express the transgene for 14–24 h, after which the cells were fixed with 4% paraformaldehyde in PEM (80 mM PIPES, 5 mM EGTA, 2 mM MgCl_2_). Cells were then permeabilized in 0.5% Triton X-100 and blocked with 5% bovine serum albumin (BSA) in Tris-buffered saline-0.1% tween-20 (TBS-T). The cells were then incubated with the appropriate primary antibodies for two hours at room temperature or overnight at 4 °C. After washing in TBS-T five times, five minutes each, secondary antibodies were applied in TBS-T for 1 h, and then cells were washed with TBS-T five additional times. Cover slips were mounted on microscope slides with Vectashield mounting medium with DAPI (Vector Labs, Burlingame, CA, USA) to visualize nuclei. The coverslips were analyzed by microscopy on a Nikon TE2000 inverted microscope, or deconvolution microscopy was performed with an Applied Precision DeltaVision image restoration microscopy with conservative deconvolution algorithms. For single cell quantification of SGs or PBs, images of 50–100 transfected cells per plasmid were scored for RNA granule content using Image J.

## 3. Results

### 3.1. PV Proteins Drive Assembly or Disassembly of SGs

**Figure 1:**
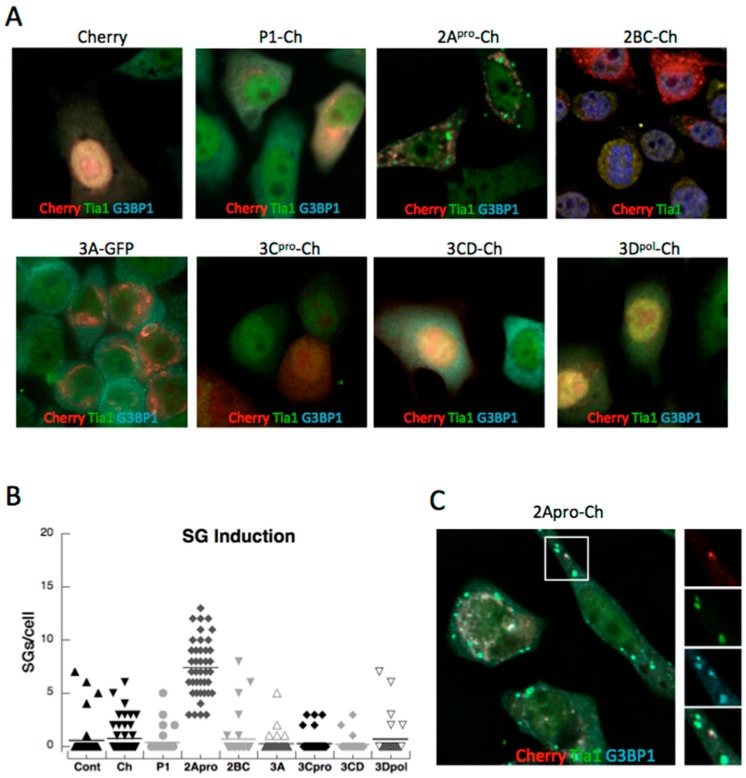
Induction of stress granules (SGs) by expression of poliovirus (PV) proteins. HeLa cells plated on glass coverslips were transfected with expression constructs for mCherry or fusions of mCherry with the indicated viral proteins. PV proteins were expressed in cells for 16 h before fixation and processing for immunofluorescence (IF) microscopy. (**A**) Representative images of cells expressing PV fusion proteins. (**B**) Box plots of the number of stress granules in individual cells expressing mCherry (Ch) or viral proteins with means indicated by horizontal lines. (**C**) 2A^pro^ appears in unique foci containing G3BP1 but not Tia1.

PV infection transiently induces SG early in infection before actively dispersing SG through cleavage of G3BP1 [14]. In order to test the contribution of other PV proteins in stimulating the stress granule response noted during infection, we generated expression constructs tagged with the mCherry fluorophore that encoded all major regions of the complete viral polypeptide, many representing completely processed PV proteins, but also some as precursors. We expressed PV-fusion proteins independently in HeLa cells and after 16 h expression examined cells for SG by immunofluorescence microscopy. Expression of the mCherry control vector did not significantly induce formation of SGs, as indicated by Tia1 and G3BP1 antibody labeling, nor did expression of the viral P1 capsid region (containing viral protein (VP)1, VP2, VP3 and VP4), 2BC, 3C^pro^, 3CD, or 3Dpolymerase (3Dpol). In contrast, PV 2A^pro^ was found to strongly induce SG formation (Figure 1). Similar results were found in A549 cells (not shown). The failure of 3C^pro^ or 3CD to induce SG was not surprising since 3C^pro^ strongly antagonizes SG formation. The finding that 2A^pro^ induced SGs was not surprising because it mediates the cleavage of host translation factor eIF4G and results in translation shutoff that generates stalled translation initiation complexes that are stored in SGs [17]. The distribution of expressed P1, 3CD, 3C^pro^ in cells was diffuse, however, 2A^pro^ was frequently found in more irregular patchy accumulations that sometimes resembled foci. Examination of foci indicated they contained G3BP1 but not Tia1, thus were not *bona fide* stress granules (Figure 1C).

**Figure 2:**
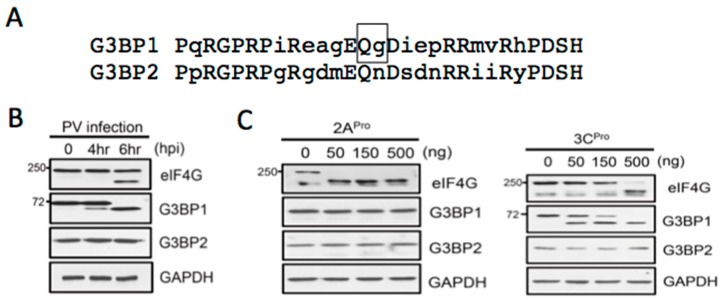
PV infection results in cleavage of G3BP1 but not G3BP2. (**A**) Sequence alignment of human G3BP1 (amino acids 311–340) and G3BP2 showing the scissile Q-G bond in G3BP1 cleaved by 3C^pro^ (box) is absent in G3BP2. (**B**) Immunoblot analysis of G3BP cleavage in PV infection. Cleavage of eIF4G1 is shown as a positive control. (**C**) *In vitro* cleavage assays using purified 2A^pro^ and 3C^pro^. Proteases were incubated with cell lysate for 60 min before assay by immunoblot.

The colocalization of G3BP1 with 2A^pro^ prompted examination of cleavage of G3BP1 and G3BP2 by both PV proteases. Potential cleavage of G3BP2 in PV infection has not been previously examined. G3BP2 is a close homolog of G3BP1 that interacts with G3BP1 and contains very high identity in the amino terminal regions and has been proposed to provide strong SG-nucleating activity and regulate some innate immune functions similar to G3BP1 [18,19]. PV 3C^pro^ substrate specificity requires an AxEQ/G motif in the P4 through P1′ positions. This motif is present in G3BP1 but the key P1′ glycine in G3BP1 (Figure 2A, box) is missing in G3BP2. This suggests G3BP2 may be refractory to cleavage at this site, however cleavage at other sites is possible. When lysates from PV-infected cells were probed for G3BP1 and G3BP2 cleavage, only the former was found cleaved (Figure 2). We further probed whether either protease could cleave G3BP1 or G3BP2 *in vitro*. As expected 3C^pro^ readily cleaved G3BP1, but G3BP2 was stable when incubated with 3C^pro^. Additionally, neither G3BP1 nor G3BP2 was cleaved by 2A^pro^. These data underscore the importance of G3BP1 cleavage by poliovirus in controlling SG assembly and raise questions concerning the functional importance of G3BP2 in SG assembly since cleavage of only G3BP1 is sufficient to destroy SG nucleation.

**Figure 3:**
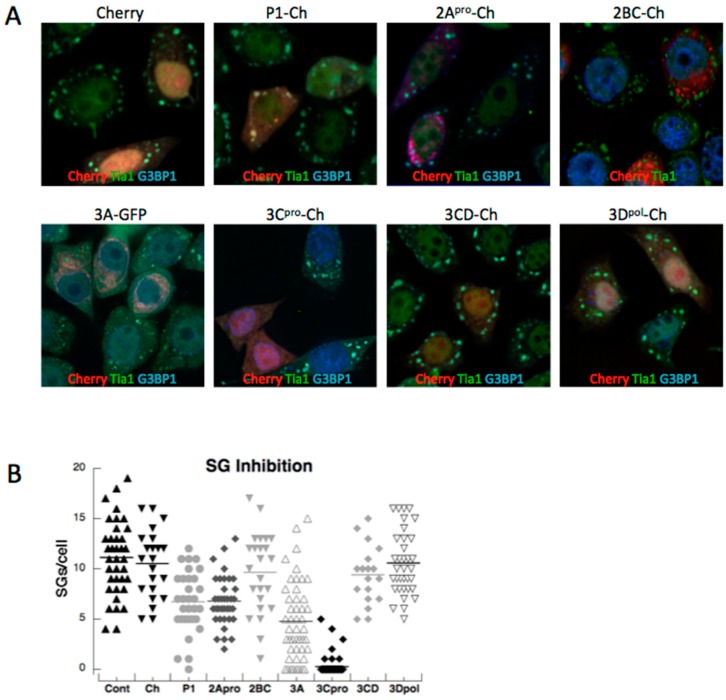
Inhibition of arsenite-induced SGs by expression of PV proteins. (**A**) Immunofluorescent microscopy of HeLa cells expressing viral proteins for 16 h and then stressed for 30 min with 200 µM sodium arsenite. (**B**) The number of stress granules/cell in individual cells is expressed in box plots.

To determine if PV 3C^pro^ was the only viral protein that blocked SG formation, cells expressing viral polypeptides were stressed with arsenite to induce SG formation and imaged by microscopy (Figure 3A). The number of SG’s per cell was determined and, as expected, it was found that expression of 3C^pro^ strongly reduced SG formation in response to arsenite. Surprisingly though, 3CD, which is the processing precursor of 3C^pro^, did not inhibit SG formation. 3CD contains efficient proteolytic activity but its substrate specificity is altered from 3C^pro^ [20,21]. In addition, viral proteins P1, 2A^pro^ and 3A displayed modest but measureable abilities to restrict SG formation when expressed in cells (Figure 3B). This suggested that other viral proteins may aide 3C^pro^ in restricting the SG assembly responses in cells.

### 3.2. Both PV Proteases Mediate PB Disruption

**Figure 4:**
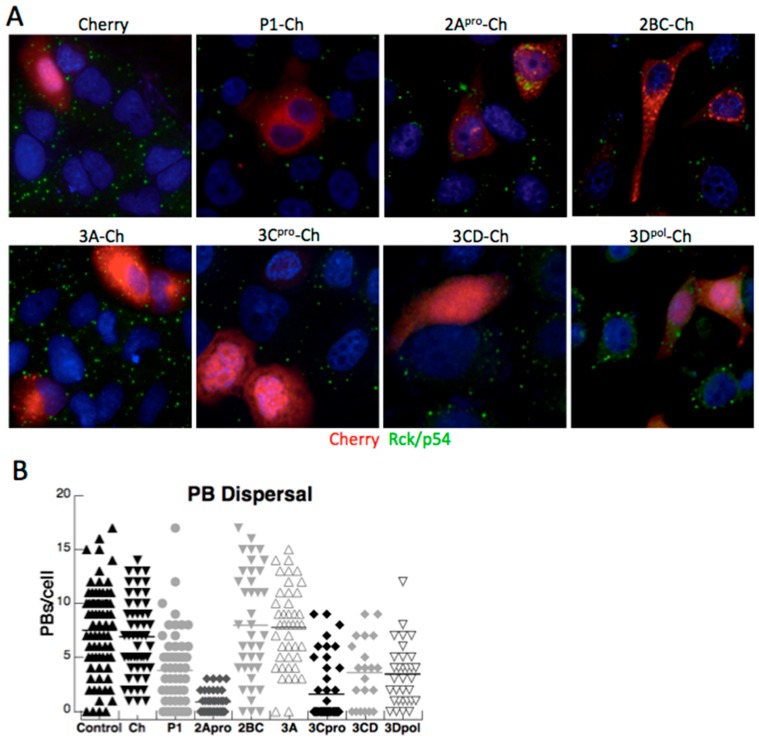
Dispersal of PBs by expression of PV proteins. (**A**) HeLa cells were transfected with expression constructs encoding mCherry, or PV virus protein fusions as indicated and expression continued for 16 h before fixation and processing for IF as described previously. Cells were incubated with an anti-Rck/p54 antibody (green) to label endogenous PBs. (**B**) The number of PBs/cell in individual cells as a measure of PB dispersal is expressed in box plots.

PV or coxsackievirus B3 infection efficiently disperses PBs in mammalian cells by mid-phase of the replicative cycle [13]. We previously linked 3C^pro^ to cleavage of PB proteins Dcp1a and PAN3, but the spectrum of viral proteins that contribute to PB dispersion and the mechanisms involved remain unclear. To determine the minimal viral proteins required for disruption of PBs we expressed the panel of viral-Cherry fusion proteins and cells were examined by microscopy for effects on PB size and abundance. Expression of the mCherry control vector did not significantly alter the size or number of constitutively present PBs, as indicated by antibody labeling of the PB marker protein Rck/p54. Similarly, expression of PV 2BC-Cherry and 3A-Cherry did not result in significant alterations in PB abundance (Figure 4). Since these proteins are primarily involved in membrane rearrangements that support viral replication, it is not surprising that they did not significantly modulate PBs, which are not membrane bound structures. In contrast, when 2A^pro^-Cherry or 3C^pro^-Cherry were expressed, endogenous PBs were significantly dispersed (Figure 4). Since 3C^pro^ mediates the cleavage of Dcp1a [13], a PB component involved in translational silencing, mRNA degradation, this was an expected result. However, 2A^pro^-mediated disruption of PB was surprising, as we have previously determined it does not cleave the three PB constituents we found that undergo degradation during infection (Xrn1, Dcp1a, or Pan3) [13]. This suggests that PV may target additional components of PBs or the pathways that control PB formation or maintenance, as 2A and 3C proteinases have distinct substrate specificity.

Expression of 3CD modestly reduced the number of PBs in cells, though not as efficiently as 3C^pro^ itself. This was in contrast to the lack effect of 3CD on SG formation noted above. Surprisingly, expression of the poliovirus polymerase (3D^pol^) had a modest inhibitory effect on PB formation.

### 3.3. Exogenous Stress can Rescue PB Formation following Chemical Dispersal

**Figure 5:**
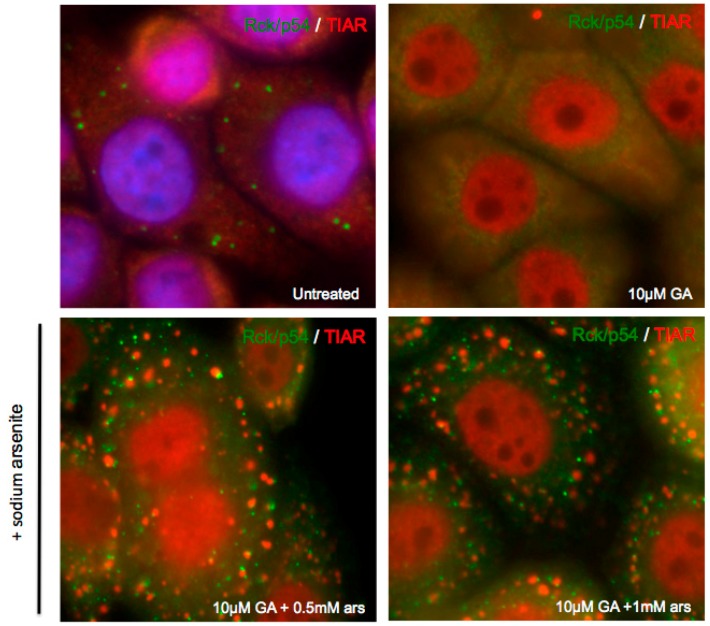
Exogenous stress restores chemically dispersed P-bodies. A549 cells were treated with 10 μM geldanamycin (GA) for 16 h and subsequently fixed for immunofluorescence or treated with sodium arsenite for 30 min prior to fixation. Cells were then incubated with antibodies against PB marker Rck/p54 (green) and SG marker TIAR (red).

Although PBs are constitutively present in cells, application of environmental stress causes PB size and number to increase in cells, likely due to the inhibition of translation and increased number of translationally silenced mRNP complexes [1]. Exogenous stress also induces the formation of SGs, which often form adjacent to PBs. Additionally, the formation of PBs requires the function of the molecular chaperone heat-shock protein 90 (Hsp90). Treatment with geldanamycin (GA), an Hsp90 inhibitor, is able to significantly disperse endogenous PBs [22,23,24]. In order to investigate the kinetics of PB induction in response to cellular stress and the connections between SGs and PBs, we utilized GA to chemically disperse the endogenous PBs of A549 cells. Subsequently, we added sodium arsenite for thirty minutes to induce oxidative stress. Strikingly, PBs were restored upon stress addition, despite the continued presence of GA at inhibitory concentrations (Figure 5). This suggested that endogenous PBs and stress-induced PBs may be compositionally different, or formed by different mechanisms, as stress-induced PBs do not require Hsp90 function.

**Figure 6:**
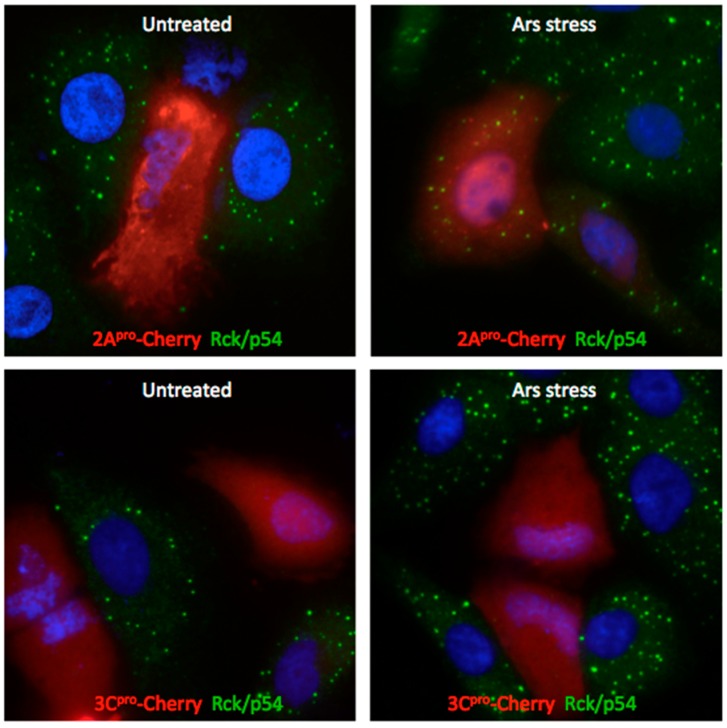
Exogenous stress rescues PBs disrupted by 2A^pro^ but not 3C^pro^. A549 cells were transfected with plasmids encoding either 2A^pro^-Cherry or 3C^pro^-Cherry and allowed to express for 16 h. Cells were then treated with 500-induced PB format for 30 min, fixed, and processed for immunofluorescence. Cells were the incubated with anti-Rck/p54 antibody (green).

### 3.4. Exogenous Stress Rescues PBs Disrupted by PV 2A^pro^ but not 3C^pro^

Since both 2A^pro^ and 3C^pro^ expression reduced the levels of endogenous PBs and we have no known targets of 2A^pro^ that associate with PBs, we explored if the proteases used a similar mechanism or targeted a common assembly pathway. We added sodium arsenite to A549 cells expressing either 2A^pro^ or 3C^pro^ Cherry fusions in an attempt to restore PB formation similarly to when PB were chemically disrupted with GA. As previously demonstrated (Figure 4), expression of either 2A or 3C proteinase significantly disperses endogenous PBs, as marked by Rck/p54. Following the addition of sodium arsenite, PB foci are rescued in cells expressing 2A^pro^ (Figure 6). This suggests that 2A^pro^ may cleave a target protein or inhibit a process involved in the maintenance or formation of constitutively expressed PB, but one that is not essential for the aggregation of stress-induced PBs. In contrast, exogenous stress does not restore PB foci in cells expressing 3C^pro^ (Figure 6), suggesting that 3C^pro^ targeted protein(s) are essential for constitutive or stress-induced PB formation. These findings strengthen the hypothesis that PV 2A and 3C proteinases have cellular targets in different PB assembly pathways.

**Figure 7:**
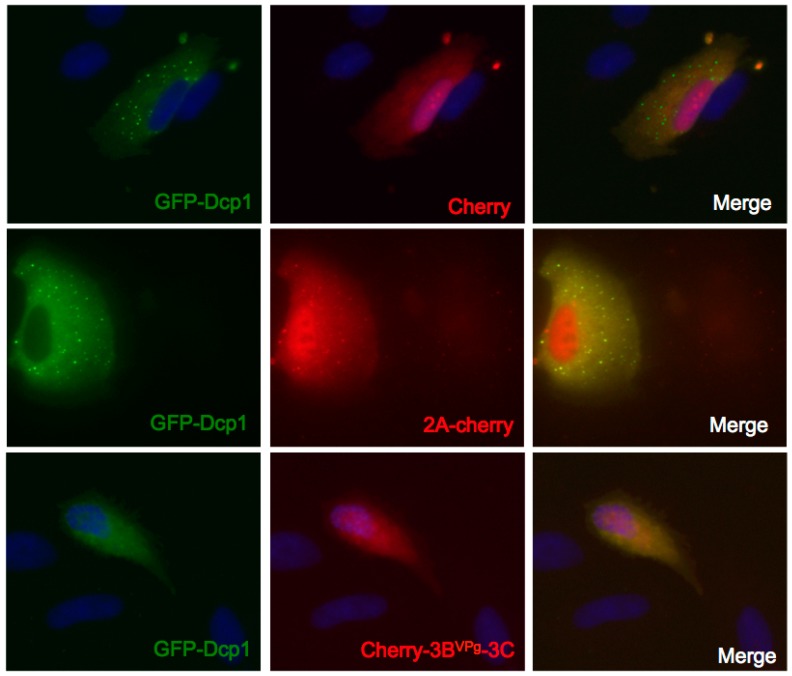
Expression of 3C^pro^, but not 2A^pro^, disrupts GFP-Dcp1a containing PB. A549 cells were co-transfected with expression vectors encoding GFP-Dcp1a (green) and mCherry, 2A^pro^-Cherry, or Cherry-3BVPg-3C^pro^ (red). After 16 h of co-expression the cells were fixed and processed for immunofluorescence.

### 3.5. Expression of 3C^pro^, but not 2A^pro^, Disrupts GFP-Dcp1a Containing PB

The decapping complex protein Dcp1a is a PB component targeted by virus infection. Previous data demonstrated that ectopic expression of Dcp1a inhibits PV translation, and that this translation repression can be relieved by the inhibition of protein kinase R (PKR) [25]. To investigate if PV proteases were capable of disrupting PBs containing ectopic GFP-Dcp1a, we co-expressed GFP-Dcp1a with plasmids encoding PV proteases. The expression of mCherry did not significantly alter the size or number of GFP-Dcp1a containing PBs in transfected cells, similar to the lack of Cherry-mediated modification of endogenous PBs (not shown). Surprisingly, expression of 2A^pro^-Cherry also did not significantly disperse GFP-Dcp1a containing PBs (Figure 7). This result is in contrast to what is observed with 2A^pro^ expression when endogenous PBs are measured (Figure 4). Since 2A^pro^ is not capable of cleaving Dcp1a, this may suggest that GFP-Dcp1a stabilizes or supplements PB assembly. In contrast, the expression of 3C^pro^ significantly disperses GFP-Dcp1a containing PBs, similarly to what is observed during PV infection when PKR is inhibited allowing viral translation (Figure 7). Two forms of 3C^pro^ were tested, one with 3C^pro^ cloned upstream of mCherry and another cloned downstream of mCherry with a cleavable VPg-linker, which gave similar results; the latter is shown. This suggests that while GFP-Dcp1a expression may prevent PB disruption by 2A^pro^, 3C^pro^ is capable of cleaving GFP-Dcp1a, as well as endogenous Dcp1a, and other factors potentially required for PB formation. This further strengthens the idea that 2A and 3C proteinases mediate the dispersal of PBs through differential mechanisms or targets.

## 4. Discussion

We have previously shown that PV blocks the formation of SGs and PBs during infection. In both cases, disruption of the RNA granules was linked to 3C^pro^ and target cellular proteins were identified in SGs (G3BP1) and PBs (Dcp1a) that are substrates of 3C^pro^ [13,14]. Though cleavage of G3BP1 is important in the viral disruption of SGs, it is possible that viral manipulation of other proteins and pathways may contribute to SG inhibition. Likewise, the role of Dcp1a cleavage in PB assembly/function has remained more enigmatic and we hypothesized that multiple viral proteins could repress PB formation. Thus, we conducted a screen of all PV gene products to determine their roles in inducing SGs or inhibiting SG and PB assembly or maintenance. Our results confirmed that PV 2A^pro^ was a strong inducer of SG assembly, similar to a previous report for CVB3 2A^pro^ [26] and that no other viral proteins induced SGs. In contrast, 2A^pro^ displayed only weak activity towards inhibition of Ars-induced SGs. However, we noted inclusion of Cherry-2A^pro^ into some unique foci that contained the SG nucleating protein G3BP, but not Tia1, indicating these foci were not *bona fide* SGs. However, the colocalization of 2A^pro^ and G3BP prompted a closer examination of the role of viral proteinases in cleavage of G3BP2. G3BP1 and G3BP2 have high homology, they interact with each other and newer reports suggest they carry out similar roles in supporting stress granule assembly [18,19,27]. Knockdown of either factor results in compensatory increased expression of the other form of G3BP in cells (not shown) and they both interact with factors ubiquitin specific peptidase 10 (USP10) and Caprin. We have not previously examined G3BP2 cleavage in infected cells as reagents were not available. G3BP2 cleavage did not occur during PV infection, and both 2A and 3C proteinases failed to cleave G3BP2 *in vitro*. Since cleavage of only G3BP1 results in such dramatic inhibition of SGs, this suggests human enteroviruses evolved to cleave the G3BP1 homolog that is functionally more important in SG assembly or innate immune signaling [28,29]. Conversely, it is possible that G3BP1 cleavage products are dominant negative inhibitors of SG assembly that can poison G3BP2-mediated assembly.

In our screen of viral factors that inhibit SG assembly, 3C^pro^ was the strongest repressor of SGs, in keeping with its role in cleaving G3BP1. Unlike 3C^pro^, the precursor 3CD, which contains proteolytic activity with altered cleavage specificity [20] had no effect on SG assembly. This suggests that G3BP1 is not cleaved by 3CD, though attempts to confirm this failed due to its toxicity and low expression level in cells. The other viral polypeptides had no strong effect on SGs, though we noted that P1 capsid proteins and 2A^pro^ displayed a modest inhibitory effect on SG assembly but did not block them.

We report here the first screen of enteroviral proteins that inhibit PB formation. PB assembly is not well understood but requires influx of partially deadenylated mRNPs, may be organized spatially by Rck/p54 and association of key RNA-decay moieties, and seems to be transiently associated with mitochondria via dynamic microtubule networks, which seem to affect the assembly of active RNA-induced silencing complex (RISC) and Ago2 association with PBs [30,31,32,33]. Surprisingly, we found in this work that 2A^pro^ blocked endogenous PB formation more effectively than 3C^pro^. No host targets of 2A^pro^ were identified previously when a series of candidate PB factors were screened that could account for disruption of PBs. This included several mRNA decapping complex components, poly(A)-nucleases, [13]. A previous report indicated 2A^pro^ cleaved Gemin 3 of the spliceosomal Sm core assembly machinery, but no other constituents [34]. These factors are not known to play roles in PBs, however Gemin 3 can complex with Ago2, a key PB component, thus an indirect role for Gemin 3 cleavage in PB assembly is possible. We also determined that expression of 3CD and even 3D^pol^ lowered average numbers of PBs in cells to about 50% normal levels. In the case of 3CD, this may be due to partial cleavage of Dcp1a or other factors. Future work will attempt to discover novel 2A^pro^, 3C^pro^ and 3CD cleavage targets that may affect PB biology.

Since there were no known cellular targets of 2A^pro^ that are thought to play roles in PB biology, we tested pathways of PB assembly using inhibitors (geldanamycin) and promoters (stress) to probe for differences in the functional outcomes imposed by each proteinase. In the current understanding of the mRNA cycle among RNA granules, mRNA cargo may enter PBs directly via mRNP remodeling in conjunction with miRNA silencing and partial poly(A) shortening or by exchange of translationally-stalled mRNP cargo from SGs, which also must involve extensive mRNPs remodeling [1,33,35]. The precise mechanism of GA-inhibition of PBs is not known, but may involve dysfunction of Argonaut proteins or GW182 [22,36] involved in miRNA loading. GA mimics ATP-binding to Hsp90 leading to Hsp90-client proteins becoming unstable and degrading [37]. Thus, in unstressed cells GA may affect loading of mRNPs via the miRNA pathway primarily.

Another mechanism promoted by GA may involve functions of eIF4E and its transporter protein 4E-T. Although most translation initiation factors only enter SGs, eIF4E and 4E-T are exceptions also found in PBs [6,23]. We found that stress could override the blockage of constitutive PB assembly imposed by geldanamycin. In these cells the percentage of PBs found interacting with SGs was unusually high, indicating a defect in mechanisms that promote cargo exchange or separation of the RNA granules. Both 2A and 3C proteinases blocked constitutive assembly of PBs, however arsenite stress rescued PBs in the presence of 2A^pro^ but not 3C^pro^. This may suggest that 2A^pro^ targets some components in the miRNA-Argonaut pathway of PB assembly, whereas 3C^pro^ targets a factor involved in the SG-mRNP cargo exchange pathway. Factors that promote SG-PB exchange are poorly defined, but may include tristetraprolin and BRF1 [1]. We note that both 2A^pro^ and 3C^pro^ cleave poly(A)-binding protein (PABP), which is a major constituent of SGs but not PBs [38,39]. PABP regulates deadenylation of mRNPs by binding Pan3 and stimulating Pan2 nuclease activity and interacting with GW182 [33,40,41,42] through a C-terminal motif that is removed by both viral proteinases. Though they affect PABP similarly, they cleave different pools of PABP in the cell, with 3C^pro^ primarily directed at translating polysome-associated PABP, whereas 2A^pro^ cleaves PABP not associated with active translation [43].

Finally, it is known that the molecular constituents of PBs differ between unstressed and stressed cells; the PBs of the latter contain YB-1, and likely other novel factors [6]. Further, overexpression of Dcp1a forms larger PBs that are resistant to dissolution with emetine, unlike endogenous PBs. Also, we have shown that expression of Dcp1a activates PKR and downstream stress pathways that block translation and will drive PB assembly [25]. Our experiments showing that only 3C^pro^ could disrupt PBs with ectopic Dcp1a is consistent with 3C^pro^, not 2A^pro^, dismantling the stress-driven pathway of PB assembly. Future work should determine the role of SG assembly itself in this pathway and to what extent PB assembly is driven by SGs under stress conditions.
